# Dual action of the cannabinoid receptor 1 ligand arachidonyl-2′-chloroethylamide on calcitonin gene-related peptide release

**DOI:** 10.1186/s10194-022-01399-8

**Published:** 2022-02-21

**Authors:** Isabella Mai Christiansen, Jacob C. A. Edvinsson, Philip V. Reducha, Lars Edvinsson, Kristian Agmund Haanes

**Affiliations:** 1 Department of Clinical Experimental Research, Glostrup Research Institute, Rigshospitalet, Glostrup, Denmark; 2 Department of Biology, University of Copenhagen, Copenhagen, Denmark; 3 Department of Drug Design and Pharmacology, Faculty of Health and Medical Sciences, University of Copenhagen, Copenhagen, Denmark; 4 Department of Medicine, Institute of Clinical Sciences, Lund University, Lund, Sweden

**Keywords:** Cannabinoid receptors, CGRP, Immunohistochemistry, Trigeminal ganglion, ACEA

## Abstract

**Background:**

Based on the current understanding of the role of neuropeptide signalling in migraine, we explored the therapeutic potential of a specific cannabinoid agonist. The aim of the present study was to examine the effect of the synthetic endocannabinoid (eCB) analogue, arachidonyl-2′-chloroethylamide (ACEA), on calcitonin gene-related peptide (CGRP) release in the dura and trigeminal ganglion (TG), as cannabinoids are known to activate G_i/o_-coupled cannabinoid receptors type 1 (CB1), resulting in neuronal inhibition.

**Methods:**

The experiments were performed using the hemi-skull model and dissected TGs from male Sprague-Dawley rats. CGRP release was induced by either 60 mM K^+^ (for depolarization-induced stimulation) or 100 nM capsaicin (for transient receptor potential vanilloid 1 (TRPV1) -induced stimulation) and measured using an enzyme-linked immunosorbent assay. The analysis of CGRP release data was combined with immunohistochemistry in order to study the cellular localization of CB1, cannabinoid receptor type 2 (CB2), CGRP and receptor activity modifying protein 1 (RAMP1), a subunit of the functional CGRP receptor, in the TG.

**Results:**

CB1 was predominantly expressed in neuronal somas in which colocalization with CGRP was observed. Furthermore, CB1 exhibited colocalization with RAMP1 in neuronal Aδ-fibres but was not clearly expressed in the CGRP-immunoreactive C-fibres. CB2 was mainly expressed in satellite glial cells and did not show substantial colocalization with either CGRP or RAMP1. Without stimulation, 140 nM ACEA per se caused a significant increase in CGRP release in the dura but not TG, compared to vehicle. Furthermore, 140 nM ACEA did not significantly modify neither K^+^- nor capsaicin-induced CGRP release. However, when the TRPV1 blocker AMG9810 (1 mM) was coapplied with ACEA, K^+^-induced CGRP release was significantly attenuated in the TG and dura.

**Conclusions:**

Results from the present study indicate that ACEA per se does not exhibit antimigraine potential due to its dual agonistic properties, resulting in activation of both CB1 and TRPV1, and thereby inhibition and stimulation of CGRP release, respectively.

**Supplementary Information:**

The online version contains supplementary material available at 10.1186/s10194-022-01399-8.

## Introduction

Migraine is a highly debilitating neurological condition linked to the sensory innervation of cranial blood vessels by the trigeminal nerves, together known as the trigeminovascular system (TVS). A pioneering study revealed that plasma levels of calcitonin gene-related peptide (CGRP), a vasodilator found in trigeminal neurons, increase significantly in craniovascular regions during the headache phase of migraine attacks, suggesting a crucial role for CGRP in migraine pathology [1].

Previous research has demonstrated CGRP release from the dura, the trigeminal ganglion (TG) and the trigeminal nucleus caudalis (TNC) in rats [2–6]. The TNC constitutes the major relay station for nociceptive afferent input from peripheral cranial structures, and is located in the brainstem in the trigeminocervical complex (TCC) [7–9]. According to the current view on migraine pathophysiology, hypothalamic activation during the premonitory phase of a migraine attack results in activation of the TNC, which in turn leads to activation of the TG. Hereafter, CGRP is released from the TG, resulting in vasodilation [10, 11].

CGRP is localized to small and medium sized (30–60 μm) neuronal somas and varicose, unmyelinated C-fibres [12], from which it is directly released [13]. C-fibres run parallel to Aδ-fibres in the TG and dura, and since Aδ-fibres are the only peripheral nerve fibres known to express the CGRP receptor (CLR/RAMP1), it has been suggested that the pain experienced by migraineurs originates from Aδ-fibres [11].

Activation of the CGRP receptor in the TG has been shown to implicate several signalling pathways known to mediate downstream physiological and pathophysiological effects. Among these is the cyclic adenosine monophosphate (cAMP) pathway, which is currently considered an antimigraine target [10, 11]. Triptans were originally developed for their vasoconstrictive properties, and later found to act by reducing intracellular cAMP levels through activation of 5-HT_1B/D_ [10]. In spite of their success, 25% of migraine patients display unresponsiveness to triptans and 40% of migraine attacks are not alleviated by triptan intervention [14], thus highlighting the need for alternative drugs for the acute treatment of migraine. Further, the contraindication of triptans for migraineurs with cardiovascular disorders prompted the development of Lasmiditan, a specific 5-HT_1F_ receptor agonist, which does not have vasoconstrictive effects [10, 15]. Although Lasmiditan does not activate 5HT_1B_, recent data suggest that part of the Lasmiditan effect might partially be through 5HT_1D_ activation [16]. The 5-HT_1B/1D/1F_ receptor subtypes are all G_i/o_-coupled G-protein coupled receptors (GPCRs) and elicit inhibitory effects on the adenylate cyclase (AC), causing a decrease in intracellular cAMP and thereby CGRP release [17]. Interestingly, the purinergic P2Y_13_ receptor, with adenosine diphosphate (ADP) as its ligand, also exhibits G_i/o_-coupling and has been shown to suppress CGRP release with results similar to that of Sumatriptan in preclinical models of migraine [5].

The endocannabinoid system (ECS) has been found in even the most primitive animals with a neuronal network, such as Hydra of the phylum Cnidaria, considered to be the first animal species possessing a neuronal network, thus illustrating the phylogenetic ancientness of the ECS [18, 19]. The two major GPCRs in the ECS are cannabinoid receptor type 1 (CB1) and cannabinoid receptor type 2 (CB2). CB1 is expressed in most brain structures as well as in the peripheral nervous system (PNS), while CB2 expression is mainly restricted to immune cells of central and peripheral tissues [20, 21]. The endocannabinoids (eCBs) 2-arachidonoylglycerol (2-AG) and N-arachidonoylethanolamine (Anandamide, AEA) are well characterized agonists of CB1 and CB2 [22].

Immunohistochemistry (IHC) on different regions of the rat brain has previously demonstrated presynaptic localization of CB1, indicating a role in regulation of neurotransmission [22, 23]. Indeed, eCBs are viewed as key regulators of synaptic function in the central nervous system (CNS). Their role in retrograde suppression of presynaptic neurotransmitter release has been well established, and studies provide evidence that presynaptic CB1 activation can lead to inhibition of neurotransmitter release (e.g., glutamate, dopamine and acetylcholine) in both human and rodent assays [24, 25]. More specifically, CB1 activation has been shown to inhibit AC activity and thereby cAMP production [26], similar to the action of triptans. In addition to activation of G_i/o_-coupled CB1 receptors, cannabinoids have also been shown to stimulate G_q/11_-coupled CB1 receptors, highlighting the complexity of cannabinoid signalling [27].

Previous studies have shown that anandamide, the endogenous non-selective cannabinoid receptor agonist, is able to inhibit trigeminovascular dilation, C- and Aδ-fibre activity as well as transient receptor potential ankyrin 1 (TRPA1) and transient receptor potential vanilloid 1 (TRPV1)-mediated CGRP release [28–31]. At higher concentrations, anandamide has also been shown to stimulate CGRP release through activation of TRPV1 [31]. The TRPV1 receptor, also known as the capsaicin receptor, has been demonstrated to colocalize with CGRP, substance P (SP) and nitric oxide (NO) in the human TG [32]. In a previous study by Fischer et al., it was examined whether methanandamide (mAEA), a synthetic anandamide analogue, modulates heat-induced CGRP release in the dura. mAEA was found to either depress (at non-noxious temperatures) or facilitate (at noxious temperatures) heat-induced CGRP release, indicating opposing dual actions of eCBs on CGRP release in the dura [33]. Since heat specifically activates TRPV1, one may ask whether eCBs exert the same dual effects on CGRP release when more general mechanisms, such as cellular depolarization, are employed to stimulate release.

Using the rat hemi-skull model, this study aims to determine the effect of arachidonyl-2′-chloroethylamide (ACEA), a synthetic anandamide analogue with high potency and selectivity for CB1 [34], on K^+^- and capsaicin-induced CGRP release from the dura and TG of male Sprague-Dawley rats. In addition, we examine the expression of CB1, CB2, receptor activity modifying protein 1 (RAMP1) and CGRP in the TG utilizing IHC.

## Methods

### Animals

Sprague-Dawley rats (300 – 400 g) purchased from Taconic (Ejby, Denmark) were maintained on a 12/12 h light/dark cycle (with dark beginning at 7 am) and housed at constant temperature (22 ± 2 °C) and humidity (55 ± 10%), with free access to food and water. Rats were generally housed in Eurostandard cages (Type VI with 123-Lid) 2–6 together.

### In situ*/*in vitro CGRP release

Rats were anaesthetized by CO_2_ inhalation and decapitated. All procedures are approved by the Danish Animal Experimentation Inspectorate. The protocol is described in detail elsewhere [35]. Following decapitation and removal of skin, skulls were immersed in synthetic interstitial fluid kept on ice to maintain metabolism at low levels. Skulls were cut mid-sagittally and brains were removed whilst the cranial dura and TGs were left attached to the hemi-skulls. The TGs were subsequently dissected from the hemi-skulls and transferred to polypropylene tubes containing 10 mL synthetic interstitial fluid (SIF) and kept in a water bath at + 37 °C for 30 min. Hemi-skulls were transferred to beakers containing SIF and placed in a water bath at + 37 °C for 15 min and then washed twice with intervals of 15 min by renewing the SIF solution.

TGs were randomized, placed in Eppendorf tubes on a heating block from Eppendorf (Hamburg, Germany) at + 37 °C, and washed five times with 300 μL SIF with intervals of 10 min. Hemi-skulls were randomized, placed on polystyrene cell culture plates from Sarstedt (Europe) in a water bath from Memmert (Bayern, Germany) at + 37 °C and washed five times with 300 μL SIF with intervals of 10 min. After the last 10 min incubation with 300 μL SIF, 200 μL samples (measuring baseline CGRP release) were extracted from the dura and TG and placed in Eppendorf tubes. The described procedures (i.e., washing and baseline extraction) were performed in all experiments prior to incubation with test compounds. It was previously demonstrated by Bhatt et al. that there is no significant difference between left and right CGRP release from the dura and TG [35], thus in the present experiments, one side functioned as a control for the other. In each experiment, one side was used for vehicle application, whereas the other was used to examine the effect of ACEA/AMG9810.

### ELISA

To measure the amount of CGRP release from both tissues (dura and TG), samples were processed using commercial CGRP (human) Enzyme Immunoassay (EIA) kits from SPIbio (Paris, France). The antibody in the EIA kit is meant for human CGRP but has 100% cross reactivity with rat CGRP [5] and was therefore utilized in this experiment. 50 μL EIA buffer was added to each of the 200 μL samples as well as to the CGRP standards. EIA buffer contains protease inhibitors and prevents CGRP degradation [5]. The CGRP tissue concentrations were determined based on the standard curve. The experiments were performed following the manufacturer’s protocol. Optical density was determined at 410 nm using a micro-plate photometer (Tecan, Infinite M200) and software from Magellan, version v.6.3 (Männedorf, Switzerland). Data was exported to Excel for analysis.

### Synthetic interstitial fluid

SIF was made using distilled water and was of the following composition (in mM): NaCl 108, KCl 3.48, MgSO_4_ 3.5, NaHCO_3_ 26, NaH_2_PO_4_ 11.7, CaCl_2_ 1.5, Na^+^ Gluconate 9.6, Glucose 5.55 and Sucrose 7.6. For K^+^-induced nerve fibre stimulation, a second SIF solution was prepared in which the KCl concentration was increased to 60 mM. To maintain similar osmolarities in the two solutions, the NaCl concentration was reduced appropriately. NaCl, NaH_2_PO_4_ and CaCl_2_ were supplied from EMSURE (Darmstadt, Germany) and the rest from Sigma-Aldrich (Darmstadt, Germany). All solutions were bubbled with 95% O_2_ and 5% CO_2_.

### Immunohistochemistry

Adult, male Sprague-Dawley rats were anaesthetized by CO_2_ inhalation and decapitated. TGs were carefully dissected and fixated with 4% paraformaldehyde (Sigma, St Louis, USA) diluted in phosphate buffered saline (PBS) for 2–4 h. To ensure cryoprotection the fixated TGs were submerged in a 10% sucrose in Sorensen’s phosphate buffer (pH 7.2) at + 4 °C for 3–4 h. Subsequently, the TGs were submerged in a 25% sucrose in Sorensen’s phosphate buffer (pH 7.2) at + 4 °C overnight. The following day, the TGs were embedded in a gelatine medium (30% egg albumin, 3% gelatine) and stored at − 20 °C. The TGs were then sectioned at 10 μm, collected on microscope slides (Superfrost, ThermoFisher) and stored at − 20 °C until used.

TG sections were thawed at room temperature (RT) and subsequently washed in PBS containing 0.25% Triton-X (PBS-T) for 2 × 15 minutes. A primary antibody was applied to each sample and the slides were incubated overnight at + 4 °C in moisturized incubation chambers. The following day, the sections were washed with PBS-T 2 × 15 minutes prior to incubation with secondary antibodies for 1 h at RT. Finally, the sections were washed 2 × 15 minutes and a coverglass was mounted with Vectashield mounting medium containing 4′,6-diamidino-2-phenylindole (DAPI) (Vector Laboratories, Burlingame CA, USA). The antibodies used and concentrations are given in Table 1.

**Table 1:** Primary and secondary antibodies used

**Product**	**Code**	**Source**	**Dilution**	**Immunogen**	**Company**
CB1	ACR-001	Rabbit	1:100	N-terminal of rat CB1 receptor	Alomone Labs, Jerusalem, Israel
CB2	ACR-002	Rabbit	1:100	3rd intracellular loop of rat CB2 receptor	Alomone Labs, Jerusalem, Israel
CGRP	Ab81887	Mouse	1:100	Rat α-CGRP	Abcam, Cambridge, UK
RAMP1	844	Goat	1:100	C-terminal of human RAMP1	Merck & Co., Inc., West Point, PA, USA
**Product**		**Source**	**Dilution**	**Against**	**Company**
FITC		Goat	1:100	Anti-rabbit	Cayman Chemical, Ann Arbor, MI, USA
Alexa 594		Goat	1:100	Anti-mouse	Jackson Immunoresearch Laboratories, Inc., West Grove, PA, USA
Cy3		Donkey	1:200	Anti-goat	Jackson Immunoresearch Laboratories, Inc., West Grove, PA, USA

For double IHC, the procedure was repeated twice before mounting. Negative controls (Supplementary Fig. 1) were performed by omitting the primary antibody. Any resulting immunofluorescence would suggest unspecific binding of the secondary antibodies or insufficient washing. The sections were examined in a light and epifluorescence microscope (Nikon 80i, Tokyo, Japan) equipped with a Nikon DS-2MV camera. Lastly, figure montages were processed in Adobe Photoshop CC20 (Adobe Systems, Mountain View, CA, USA).

### Compounds

ACEA, supplied pre-dissolved in anhydrous ethanol (5 mg/mL, 13.66 mM), was purchased from Tocris (Bristol, UK). Ethanol was used as vehicle for ACEA throughout all experiments and did not exceed 0.001%. Capsaicin was supplied from Tocris (Bristol, UK), dissolved in a 10 mM stock solution in 100% ethanol and was further diluted to a 0.001% ethanol containing solution of 100 nM capsaicin. AMG9810 was supplied from Tocris (Bristol, UK) and was dissolved at 1 mM in a 10% ethanol and 90% DMSO containing solution, which was also used as vehicle. When diluted the concentration of ethanol and DMSO was 0.01% and 0.09%, respectively. All remaining compounds were purchased from Sigma Aldrich/Merck (Darmstadt, Germany).

### Statistics

All data was subject to Grubb’s test (α = 0.05) which was performed using the GraphPad outlier calculator. All the CGRP and Substance P release data passed the Shapiro-Wilk normality test (α = 0.05). Further, all identified outliers were removed from the dataset prior to performing Student’s T-test and calculating mean and SEM values. Data for CGRP release is shown with mean ± SEM, where ‘n’ represents the sample size (i.e., number of animals).

## Results

### CGRP release

It has been well established in both human and rodent assays that G_i/o_-coupled CB1 receptors are involved in retrograde suppression of neurotransmitter release [24, 25]. Since ACEA is a potent and highly selective CB1 agonist [34], we sought to examine the therapeutic potential of ACEA in migraine pathology by investigating whether ACEA per se*,* affects CGRP levels in the dura and TG of male Sprague-Dawley rats.

**Fig. 1:**
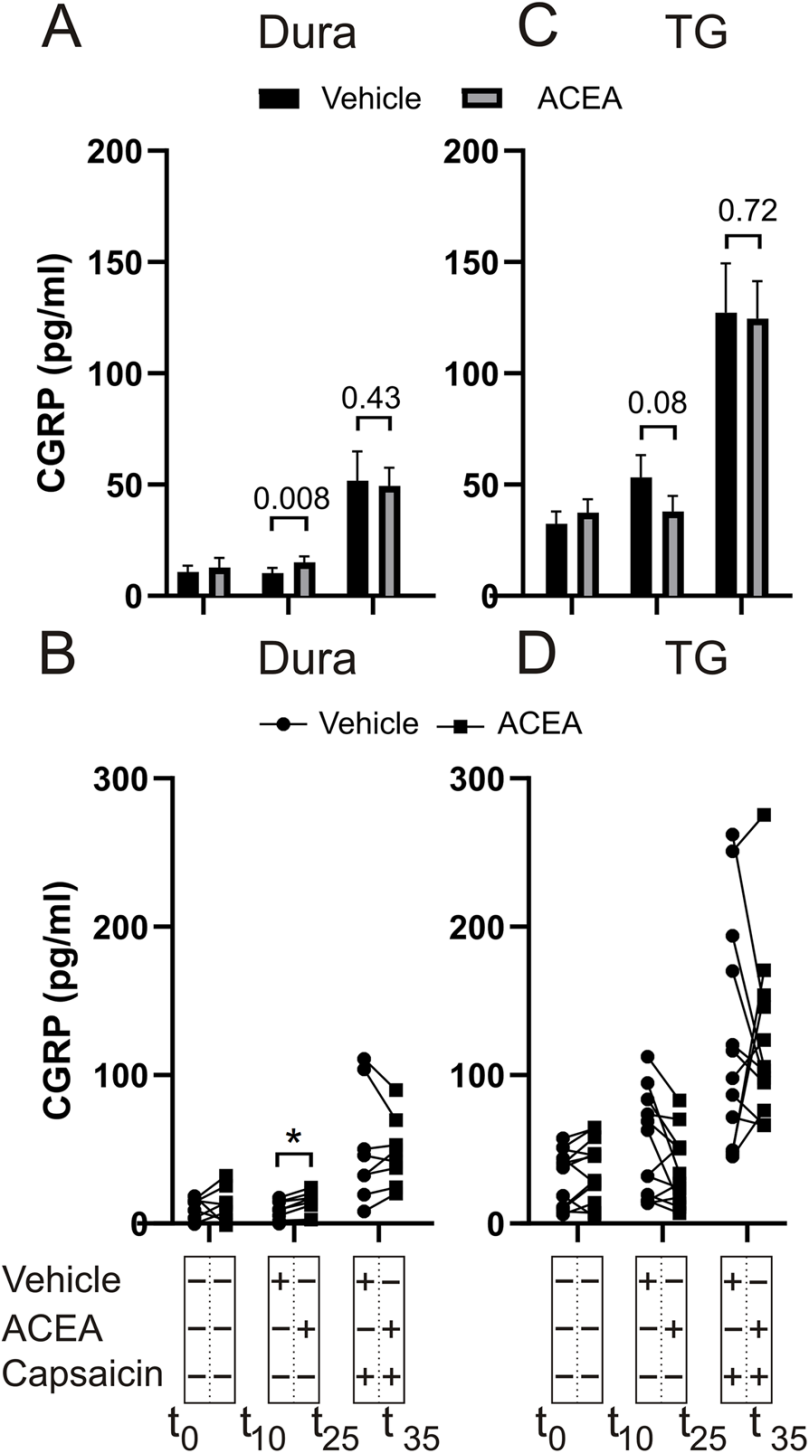
Effects of ACEA on capsaicin-induced CGRP release from dura and TG. Application of 140 nM ACEA caused a significant increase in CGRP release from the dura (*n* = 8). 100 nM capsaicin caused a significant increase in CGRP release, which was not attenuated by the presence of ACEA. **B** Graphical illustration of the sample pairing. **C** Application of 140 nM ACEA had no effect on the CGRP release from the TG per se, nor on the release induced by 100 nM capsaicin (*n* = 12). **D** Graphical illustration of the sample pairing. B = baseline. Timeline in minutes are added on the lower panel. Data are shown as mean ± SEM or their individual data points with pairing, and with *p* values obtained with Student’s T-test being depicted in the graph (* = *p* < 0.05). For reference 100 pg/ml equals 26.4 pmol/l

140 nM ACEA per se caused a significant increase in CGRP release from the dura (w/o ACEA: 10.3 ± 2.2 pg/mL; w/ACEA: 15.0 ± 2.8 pg/mL; *p* = 0.008, *n* = 8) compared to vehicle (Fig. 1A-B). In the TG however, the difference was non-significant (w/o ACEA: 53.3 ± 10.1 pg/mL; w/ACEA: 37.9 ± 7.1 pg/mL, *p* = 0.08, *n* = 12) (Fig. 1C-D). In continuation, we performed an experiment in which 100 nM capsaicin was used as a TRPV1-specific stimulus (Fig. 1). 140 nM ACEA did not cause significant inhibition of capsaicin-induced CGRP release in the TG (w/o ACEA: 127.2 ± 22.1 pg/mL; w/ACEA: 124.6 ± 16.9 pg/mL, *p* = 0.72, *n* = 12) or dura (w/o ACEA: 51.7 ± 13.3 pg/mL; w/ACEA: 49.4 ± 8.2 pg/mL, *p* = 0.43, *n* = 8) (Fig. 1). Considering that ACEA is the synthetic analogue of anandamide, which has been shown to stimulate CGRP release through activation of TRPV1 [31], it is possible that ACEA stimulated CGRP release through a higher degree of TRPV1 than CB1 activation.

**Fig. 2:**
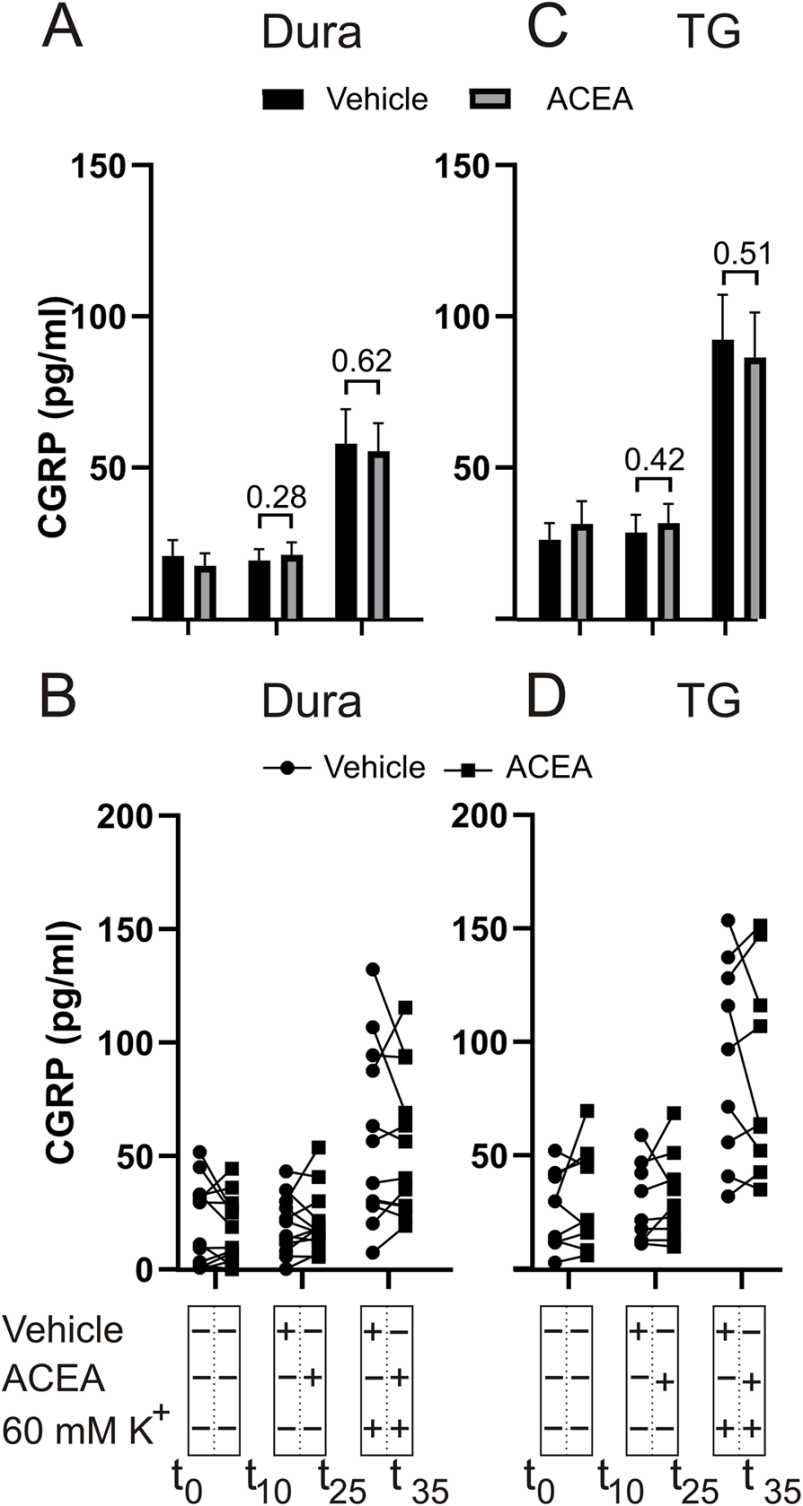
Effects of ACEA on K^+^-induced CGRP release from dura and TG. **A** Application of 140 nM ACEA did not cause a significant increase in CGRP release from the dura (*n* = 12). KCl (60 mM K^+^) caused a significant increase in CGRP release, which was not attenuated by the presence of ACEA. **B** Graphical illustration of the sample pairing. **C** Application of 140 nM ACEA had no effect on the CGRP release from the TG per se, nor on the release induced by 60 mM K^+^ (*n* = 12). **D** Graphical illustration of the sample pairing. B = baseline. Timeline in minutes are added on the lower panel. Data are shown as mean ± SEM or their individual data points with pairing, and with *p* values obtained with Student’s T-test being depicted in the graph (* = *p* < 0.05). For reference 100 pg/ml equals 26.4 pmol/l

Due to the potential implication of TRPV1 in CGRP release, we performed another experiment in which 60 mM K^+^ was used as depolarizing stimulus. Addition of 140 nM ACEA per se showed a slight but similar tendency to stimulate CGRP release in the dura (w/o ACEA: 19.4 ± 3.7 pg/mL; w/ACEA: 21.2 ± 4.0 pg/mL, *p* = 0.28, *n* = 12) as well as in the TG (w/o ACEA: 28.6 ± 5.9 pg/mL; w/ACEA: 31.7 ± 6.4 pg/mL, *p* = 0.42, *n* = 9) compared to vehicle (Fig. 2). Further, due to the difference observed in these experiments, we combined all data after adding ACEA and compared to vehicle. The direct effect of adding ACEA per se was a change from baseline of 4.1 ± 1.9 pg/mL compared to vehicle at − 0.7 ± 1.7 pg/mL being significant (*p* = 0.023, *n* = 20). When we further tested whether the addition of 140 nM ACEA would affect K^+^-induced CGRP release, no significant inhibition was observed in the dura (Fig. 2A-B) or TG (Fig. 2C-D).

**Fig. 3:**
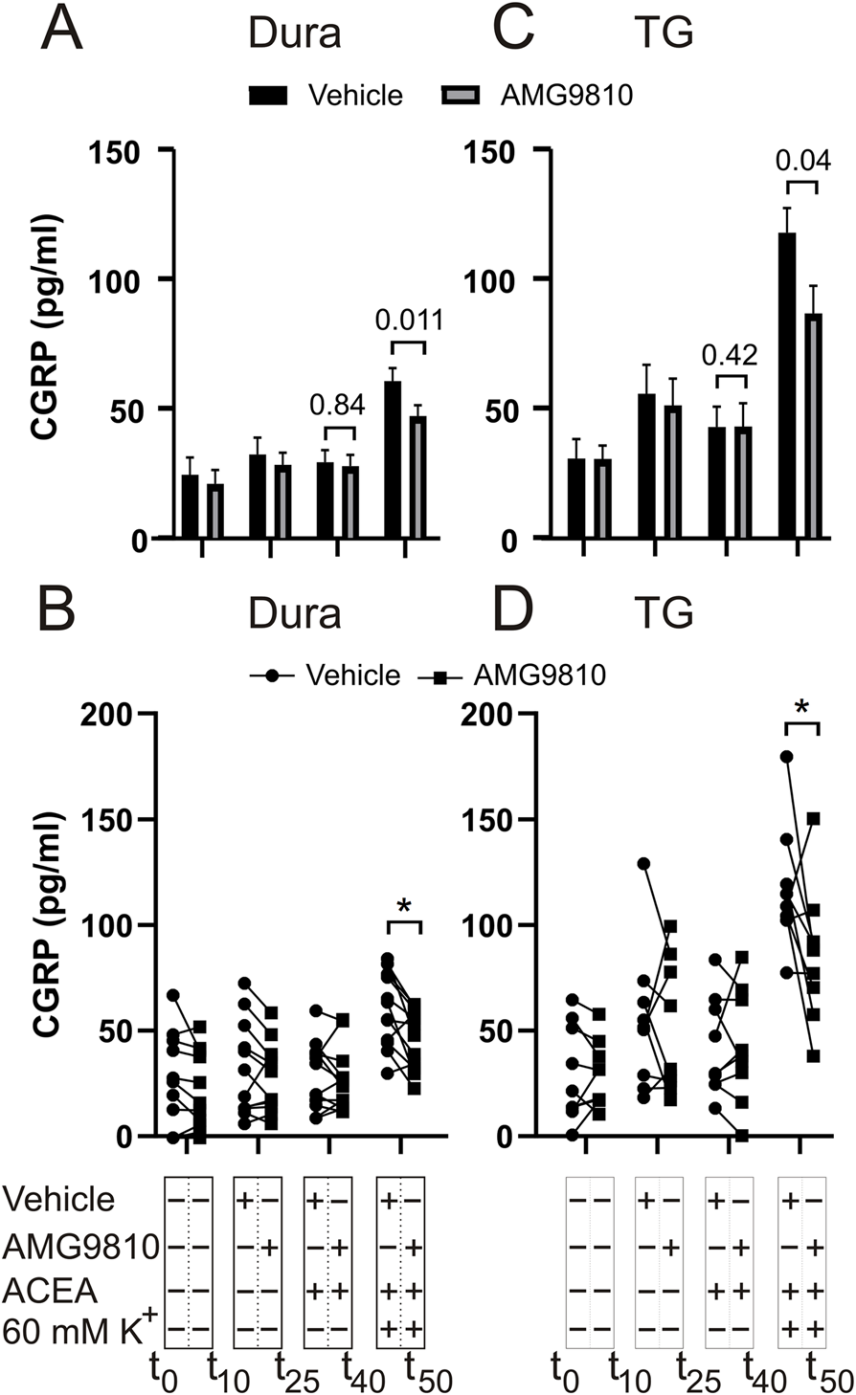
Effects of ACEA on K^+^-induced CGRP release from dura and TG in the presence of AMG9810. **A** Application of 140 nM ACEA in the presence of AMG9810, did not cause a significant increase in CGRP release from the dura (*n* = 12). KCl (60 mM K^+^) caused a significant increase in CGRP release, which in the presence of 1 μM AMG9810 was significantly attenuated by ACEA. **B** Graphical illustration of the sample pairing. **C** Application of 140 nM ACEA had no effect on the CGRP release from the TG per se, but significantly inhibited the release induced by 60 mM K^+^ (*n* = 12) in the presence of 1 μM AMG9810. **D** Graphical illustration of the sample pairing. B = baseline. Timeline in minutes are added on the lower panel. Data are shown as mean ± SEM or their individual data points with pairing, and with p values obtained with Student’s T-test being depicted in the graph (* = *p* < 0.05). For reference 100 pg/ml equals 26.4 pmol/l

Since no significant inhibition was observed using K^+^ as depolarizing stimulus, it was considered whether TRPV1 competes with CB1 for binding to ACEA. Therefore, we performed an experiment using AMG9810, a potent and selective, competitive antagonist of TRPV1. Upon K^+^-stimulation in the presence of 1 mM AMG9810, 140 nM ACEA demonstrated significant inhibition of CGRP release in the dura (w/o AMG9810: 60.5 ± 5.1 pg/mL; w/AMG9810: 47.2 ± 4.1 pg/mL, *p* = 0.011, *n* = 12) (Fig. 3A-B) and TG (w/o AMG9810: 117.7 ± 9.6 pg/mL; w/AMG9810: 86.6 ± 10.6 pg/mL, *p* = 0.04, *n* = 9) (Fig. 3C-D) compared to ACEA w/o AMG9810.

### Immunohistochemistry

**Fig. 4:**
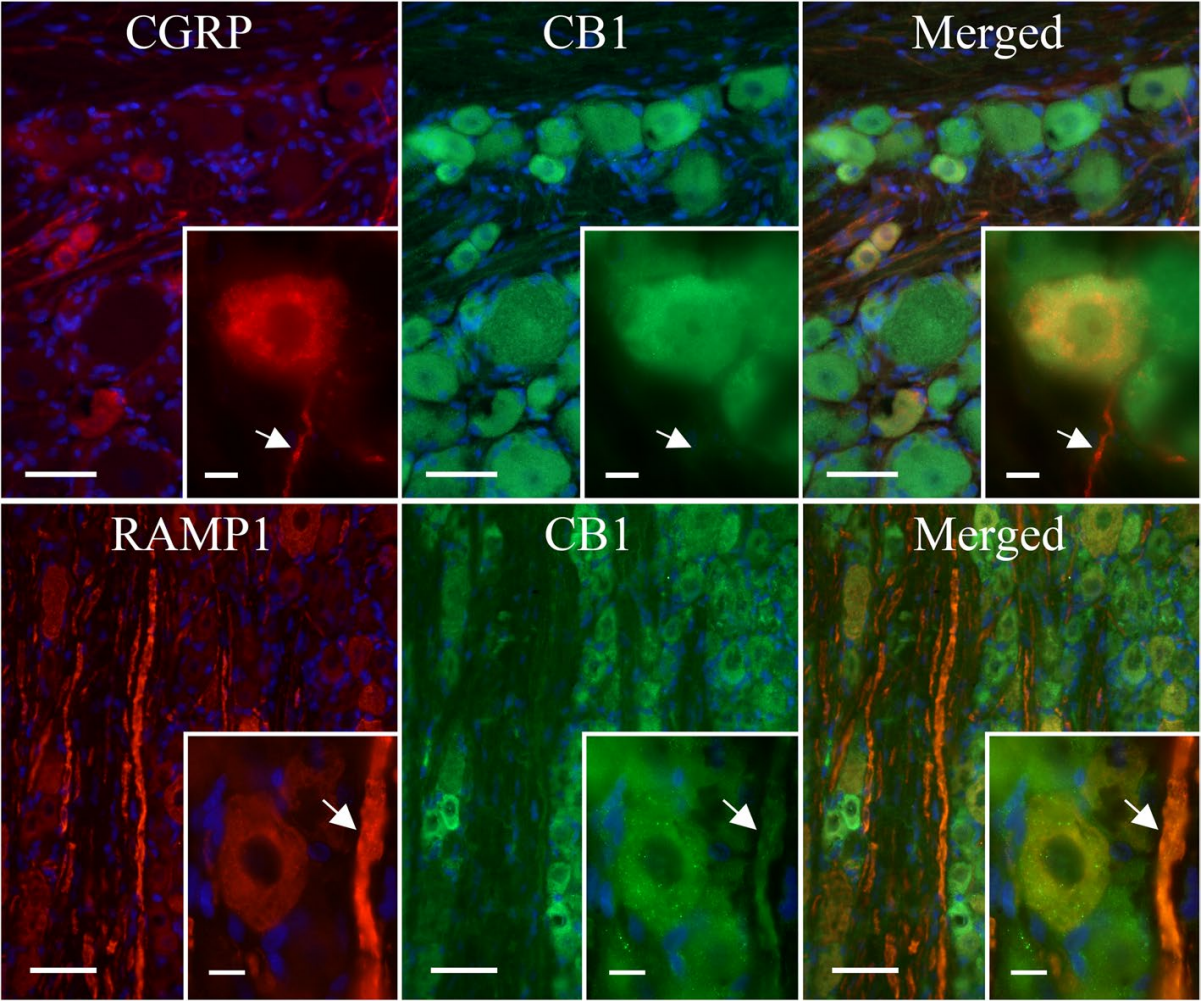
The expression of CB1, CGRP and RAMP1 in the TG. IHC localization of CB1, CGRP and the functional CGRP receptor (by staining for RAMP1) in the TG. Blue represents cell nuclei; orange represents colocalization of CGRP (upper panel) or RAMP1 (lower panel) with CB1; globular regions represent neuronal somas and fibrous regions represent individual neuronal fibres. Large image scale corresponds to 50 mm, and the insert scale corresponds to 10 μm. The arrows mark unmyelinated C-fibres (upper panel) and myelinated Aδ-fibres (lower panel)

IHC images were used to determine the cellular location of CB1, CB2, CGRP and RAMP1 in the TG. RAMP1 is an essential part of the functional CGRP receptor [36] and therefore represents potential localization of the CGRP receptor, although it could also label neurons with the Amylin 1 receptor [37]. Nevertheless, Fig. 4 shows a high expression level of CB1 in neuronal somas of the TG relative to that observed in satellite glial cells (SGCs) and neuronal fibres. In contrast, CGRP was highly expressed in C-fibres with limited localization to neuronal somas of the TG (Fig. 4A), whereas RAMP1 exhibited high expression levels in both Aδ-fibres and neuronal somas (Fig. 4B). Furthermore, CB1 was found to colocalize with CGRP in neuronal somas, whereas colocalization in the CGRP associated C-fibres was not clearly detected (Fig. 4A). Colocalization of CB1 and RAMP1 was observed in neuronal somas and Aδ-fibres of the TG (Fig. 4B).

**Fig. 5:**
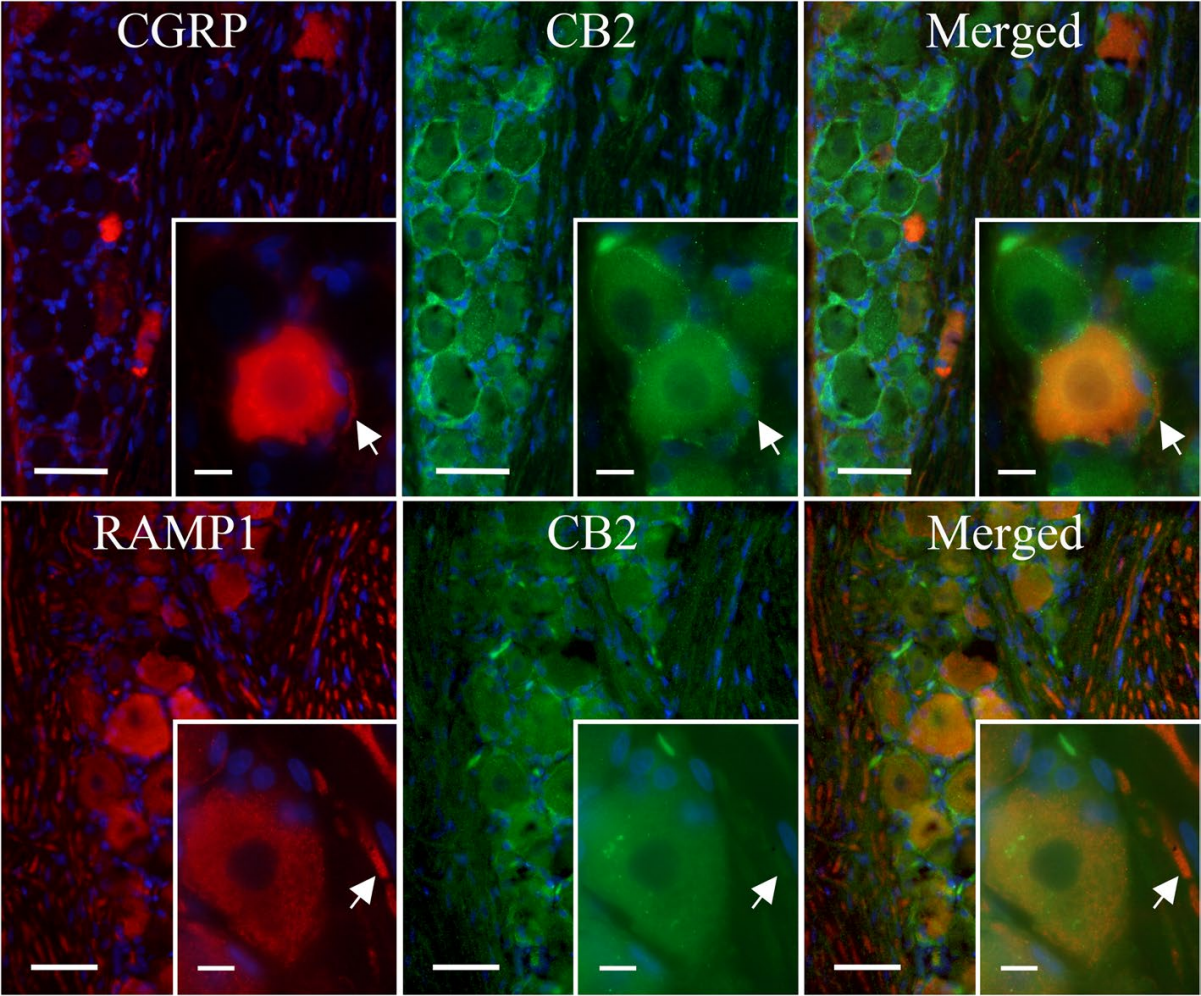
The expression of CB2, CGRP and RAMP1 in the TG. IHC localization of CB2, CGRP and the functional CGRP receptor (by staining for RAMP1) in the TG. Blue represents cell nuclei; orange represents colocalization of CGRP (upper panel) or RAMP1 (lower panel) with CB2; globular regions represent neuronal somas and fibrous regions represent individual neuronal fibres. Large image scale corresponds to 50 mm, and the insert scale corresponds to 10 μm. The arrows mark unmyelinated C-fibres (upper panel) and myelinated Aδ-fibres (lower panel)

CB2 was found to be highly expressed in many SGCs relative to that observed in neuronal somas and fibres of the TG (Fig. 5). In addition, CB2 did not demonstrate colocalization with neither CGRP (Fig. 5A) nor RAMP1 (Fig. 5B) in the TG, supporting the notion that ACEA exerts its effect through CB1, and not CB2.

## Discussion

The data presented in the present study shows that the cannabinoid system in the TVS must be seen in light of TRPV1 crosstalk. CB1 activation is able to reduce CGRP release, but only when TRPV1 is blocked. This adds to the notion that receptors which couple negatively through cAMP have antimigraine potential, but off target effects could hide such responses.

IHC results from the TG revealed abundant expression of CB1 in neuronal somas, and to a lesser extent in C- and Aδ-fibres (Fig. 4). In addition, IHC results showed colocalization of CB1 with CGRP and RAMP1 (an indicator of CGRP receptor expression) in neuronal somas and to some extent in fibres of the TG, supporting the notion that CB1 may act through retrograde feedback inhibition of CGRP. We cannot rule out a possible interaction with neurons containing the Amylin 1 receptors, which has been detected in TG [38, 39]. Our findings are in agreement with those of Price et al. which demonstrate predominant colocalization of CB1 with markers for myelinated large diameter fibres in the TG, and to a lesser extent with CGRP containing small diameter fibres [40]. In addition, Price et al. show that CB1 rarely colocalizes with TRPV1 in the TG [40], suggesting that CB1 and TRPV1 do not act in a concerted manner, but rather as part of two distinct systems both governing modulation of nociception and CGRP signalling.

Looking at expression data of the receptors, we re-analysed previously published data that was constructed using publicly available sequencing data generated by Hougaard Pedersen and collaborators [41]. RNA counts are depicted relative to 5-HT_1B_, since 5-HT_1B_ agonists are the gold standard in migraine treatment and have shown to effectively inhibit cAMP production and CGRP release [17]. As can be seen in Supplementary Fig. 2 the average CB1 expression level is considerably higher than that of 5-HT_1B/1D_, CB2 expression only constitutes approximately 10% of 5-HT_1B/1D_ expression. Furthermore, it was observed that TRPV1 is expressed almost double that of CB1 in the TG, roughly corresponding to an expression level 10 times that of 5-HT_1B/1D_.

**Fig. 6:**
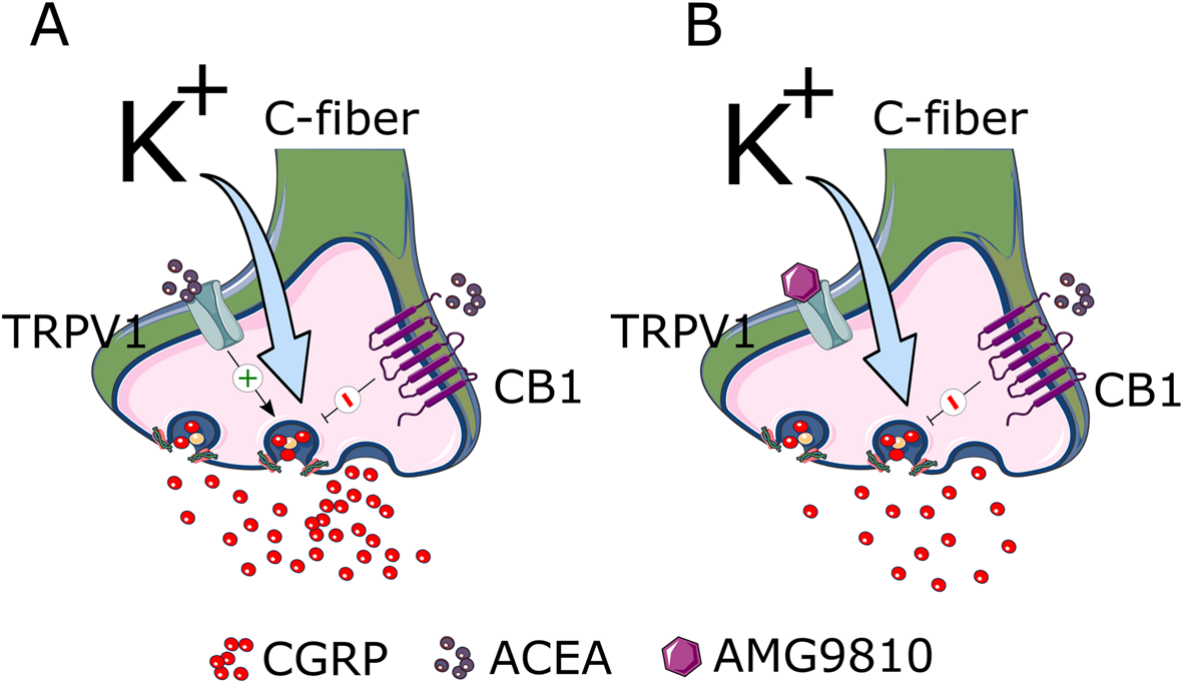
Graphical illustration of the interaction of CB1 and TRPV1 by cannabinoids. **A** ACEA activates both TRPV1 and CB1 in a native setting, leading to no observed inhibition. **B** when TRPV1 is inhibited, activation of CB1 leads to an attenuation of the CGRP release

The initial application of ACEA demonstrated a significant increase in CGRP release from the dura, whereas the response from the TG was non-significant. Although ACEA is a relatively specific agonist of CB1, it has been shown to activate TRPV1 [42]. When AMG9810, a blocker of TRPV1, was added (Fig. 3) there was no CGRP release caused by ACEA, suggesting that the effect was mediated by TRPV1 activation. This indicates that the slight tendency of ACEA to augment the capsaicin-induced response was caused by additional activation of TRPV1, and that the postulated ACEA-mediated CB1 activation did not constitute the main effect. The non-significant response observed in the TG upon ACEA application may be explained by the dual action of ACEA acting on G_i/o_-coupled CB1 receptors as well as on TRPV1 to inhibit and stimulate CGRP release, respectively, leading to a balanced response (Fig. 6).

When using K^+^-induced depolarization as stimulation, we hypothesized that CB1 activation and subsequent inhibition of CGRP release would be the predominant pathway. This was based on the earlier notion that anandamide is able to inhibit K^+^ (and capsaicin)-induced CGRP release [43]. However, no significant inhibition was observed in this setting, although a tendency of inhibition was seen in the TG (Fig. 2). There is contrasting evidence from previous studies of the involvement of CB1 in CGRP release, particularly in relation to TRPV1 activation. This leads to a discussion of the interaction between the cannabinoid and TRPV1 systems. Several arachidonic acid metabolites, including anandamide, are viewed as putative endogenous TRPV1 ligands, termed endovanilloids [44]. Interestingly, many of the transient receptor potential (TRP) channels have been considered “ionotropic cannabinoid receptors” [45], and anandamide has been shown to stimulate CGRP release through additional activation of TRPV1 [31]. Somewhat paradoxical, eCBs would therefore be able to: i) stimulate CGRP release through activation of TRPV1, and ii) inhibit CGRP release through activation of G_i/o_-coupled CB1 receptors or by homologous and heterologous desensitization of TRPV1 and TRPA1, respectively [30, 31]. Indeed, Akerman and colleagues have suggested that in the TCC or TNC, anandamide inhibits neurons with C-fibre input through activation of CB1, an inhibition which is increased after blockade of TRPV1, [29] and Messlinger and colleagues have indicated opposing dual actions of eCBs on CGRP release in the dura [33]. This contrasts with the actions of anandamide in the periphery where the primary effect is postsynaptic inhibition of dural blood vessel dilatation, and only partial inhibition of CGRP release [28]. Current knowledge on how and which cannabinoids target TRP channels is still scarce [46]. It appears that ACEA adds to the eCB analogues that previously have been shown to activate TRPV1 [47], and in our setup can cause CGRP release.

In comparison to other systems, several neurons in the dorsal periaqueductal grey (dPAG) co-express CB1 and TRPV1, making it possible that an endogenous substance, i.e., anandamide, may exert both panicolytic and panicogenic effects via its actions at CB1 and TRPV1, respectively [48]. It has been postulated by Tognetto and colleagues that lower anandamide levels could act mainly on CB1, whereas TRPV1-mediated actions may prevail over CB1 at higher concentrations [49]. We are therefore not the first to suggest that if large amounts of endogenous anandamide could be produced, that could activate, rather than inhibit, nociceptive sensory neurons [49]. In support of this, it has previously been shown that anandamide application results in excitation of peripheral terminals of capsaicin-sensitive primary sensory neurons via cannabinoid receptor independent mechanisms, most likely TRPV1 activation, and causes CGRP release [50]. Further, Tognetto and colleagues showed that application of higher concentrations of anandamide to capsaicin-sensitive dorsal root ganglia (DRG) neurons leads to neuropeptide release [49]. It has long been known that the endogenous ligand anandamide behaves as an agonist of TRPV1 [51]. One major contrast between the cannabinoid- and capsaicin-evoked currents for TRPV1 is the lack of apparent pore dilation (a characteristic of a two state channel) in response to cannabinoids, even when large currents are driven by high doses of cannabinoids or long induction times [52]. However, the in vivo effects of this are not well understood. Interestingly, this cross activation of TRPV1 also seems to apply to the more CB1-specific ACEA, as we in the current study were not able to find a window of pure inhibition per se. Thus, we conclude that cannabinoid signalling in the TG shows similarities to other systems and appears to be complexly regulated.

What effect does CB1 activation exert when TRPV1 is blocked? AMG9810, which is a potent and competitive TRPV1-selective antagonist [53], was administered prior to ACEA in order to determine whether the ACEA-induced increase in CGRP levels without stimulation was a result of TRPV1 activation. Unlike capsazepine, which only inhibits the capsaicin-induced Ca^2+^ influx, AMG9810 inhibits capsaicin-, proton-, heat- and endogenous agonist-induced activation of TRPV1 [53], thus presenting a broader spectrum of inhibition. When TRPV1 was blocked, CB1 activation had inhibitory effects both in the dura and in the TG (Fig. 6). It has been well established in both human and rodent assays that G_i/o_-coupled CB1 receptors are involved in retrograde suppression of neurotransmitter release [24, 25]. We therefore conclude that CB1 activation leading to cannabinoid-mediated inhibition of CGRP release only occurs when TRPV1 is blocked. This supports the findings by Akerman et al., in which anandamide inhibited A-fibre inputs to the TCC only in the presence of capsazepine [29], as well as the findings by Fischer et al., showing the dual actions of eCBs on heat activated CGRP release [33], highlighting the dual agonistic properties of cannabinoids throughout the TVS and with various stimuli.

In the current context, a study by Summ et al. demonstrated that application of TRPV1 antagonist A-993610 did not per se inhibit C- and Aδ-fibre activity in the TCC in response to electrical stimulation of the middle meningeal artery or after spreading depression [54]. Therefore it does not appear that TRPV1 antagonism in itself is a sufficient migraine target, but the data presented here show that it could potentially be combined with other drugs targeting the eCB system. Finding the right dose combination, which stimulates CB1 without activating TRPV1, would need more complex pharmacokinetics. Nevertheless, our study confirms and further support the hypotheses [10, 55] that receptors which lower the cAMP levels are potential pharmacological targets for treating migraine. Our study highlights the need to preclinically test for any off-target effects.

## Conclusion

In conclusion, application of ACEA alone significantly stimulated CGRP release from the dura, indicating that ACEA does not exhibit antimigraine potential, at least based on data from the current setup. Cannabinoid signalling pathways appear to be very complex with many factors, such as functional selectivity, agonist concentration and regional differences in protein distribution, capable of modulating the response. We show that CB1 could have antimigraine potential when TRPV1 is blocked, but that the clinical applicability of this finding might be limited. Although the physiological activation of TRPV1 most likely is heat activation, we suggest that future studies should consider that TRPV1 channel activation could be due to a pathological response during overactive cannabinoid signalling.

## Supplementary Information

**Additional file 1: Supplementary Fig. 1.** Negative control for CB1 and CB2. The negative control for the FITC antibody used for CB1 and CB2 IHC.

**Additional file 2: Supplementary Fig. 2.** mRNA expression in the trigeminal ganglion. The average RNA counts of 10 rats in the TG. RNA counts are depicted on a logarithmic scale relative to 5-HT_1B_ and are shown for 5-HT_1B_; 5-HT_1D_; CB1; CB2; TRPV1; RAMP1 and CGRP.

## Data Availability

The datasets generated during and/or analysed during the current study are available from the corresponding author on reasonable request.

## Abbreviations

ADP: Adenosine Diphosphate
AC: Adenylate Cyclase
Anandamide: N-arachidonoylethanolamine
ACEA: Arachidonyl-2′-chloroethylamide
CGRP: Calcitonin Gene-Related Peptide
CB1: Cannabinoid Receptor Type 1
CB2: Cannabinoid Receptor Type 2
cAMP: Cyclic Adenosine Monophosphate
CNS: Central Nervous System
dPAG: Dorsal Periaqueductal Gray
DRG: Dorsal Root Ganglia
eCB: Endocannabinoid
ECS: Endocannabinoid system
ELISA: Enzyme-Linked Immunosorbent Assay
GPCR: G-Protein Coupled Receptor
GTN: Nitro-glycerine
IHC: Immunohistochemistry
MMA: Middle Meningeal Artery
NO: Nitric Oxide
PBS: Phosphate Buffered Saline
PNS: Peripheral Nervous system
RAMP1: Receptor Activity Modifying Protein 1
RT: Room Temperature
SGC: Satellite Glial Cell
SIF: Synthetic Interstitial Fluid
SP: Substance P
TRP: Transient Receptor Potential
TRPA1: Transient Receptor Potential Ankyrin 1
TRPV1: Transient Receptor Potential Vanilloid 1
TG: Trigeminal Ganglion
TNC: Trigeminal Nucleus Caudalis
TCC: Trigeminocervical Complex
TVS: Trigeminovascular System
TRP: Transient Receptor Potential
2-AG: 2-arachidonoylglycerol
5-HT: 5-hydroxytryptamine

## References

[1] Goadsby PJ, Edvinsson L, Ekman R (1990). Vasoactive peptide release in the extracerebral circulation of humans during migraine headache. Ann Neurol.

[2] Ebersberger A, Averbeck B, Messlinger K, Reeh PW (1999). Release of substance P, calcitonin gene-related peptide and prostaglandin E2 from rat dura mater encephali following electrical and chemical stimulation in vitro. Neuroscience.

[3] Amrutkar DV, Ploug KB, Hay-Schmidt A, Porreca F, Olesen J, Jansen-Olesen I (2012). mRNA expression of 5-hydroxytryptamine 1B, 1D, and 1F receptors and their role in controlling the release of calcitonin gene-related peptide in the rat trigeminovascular system. Pain.

[4] Edvinsson JCA, Grell A, Warfvinge K, Sheykhzade M, Edvinsson L, Haanes KA (2020). Differences in pituitary adenylate cyclase-activating peptide and calcitonin gene-related peptide release in the trigeminovascular system. Cephalalgia.

[5] Haanes KA, Labastida-Ramirez A, Blixt FW, Rubio-Beltran E, Dirven CM, Danser AH (2019). Exploration of purinergic receptors as potential anti-migraine targets using established pre-clinical migraine models. Cephalalgia.

[6] Edvinsson JC, Reducha PV, Sheykhzade M, Warfvinge K, Haanes KA, Edvinsson L (2021). Neurokinins and their receptors in the rat trigeminal system: Differential localization and release with implications for migraine pain. Mol Pain.

[7] Goadsby PJ, Holland PR, Martins-Oliveira M, Hoffmann J, Schankin C, Akerman S (2017). Pathophysiology of migraine: a disorder of sensory processing. Physiol Rev.

[8] Bartsch T, Goadsby PJ (2003). The trigeminocervical complex and migraine: current concepts and synthesis. Curr Pain Headache Rep.

[9] Edvinsson JCA, Vigano A, Alekseeva A, Alieva E, Arruda R, De Luca C (2020). The fifth cranial nerve in headaches. J Headache Pain.

[10] Haanes KA, Edvinsson L (2019). Pathophysiological mechanisms in migraine and the identification of new therapeutic targets. CNS Drugs.

[11] Edvinsson L, Haanes KA (2021). Identifying new antimigraine targets: lessons from molecular biology. Trends Pharmacol Sci.

[12] Eftekhari S, Salvatore CA, Johansson S, Chen TB, Zeng Z, Edvinsson L (2015). Localization of CGRP, CGRP receptor, PACAP and glutamate in trigeminal ganglion. Relation to the blood-brain barrier. Brain Res.

[13] Edvinsson JCA, Warfvinge K, Krause DN, Blixt FW, Sheykhzade M, Edvinsson L (2019). C-fibers may modulate adjacent Adelta-fibers through axon-axon CGRP signaling at nodes of Ranvier in the trigeminal system. J Headache Pain.

[14] Diener HC, Dodick DW, Goadsby PJ, Lipton RB, Almas M, Parsons B (2008). Identification of negative predictors of pain-free response to triptans: analysis of the eletriptan database. Cephalalgia.

[15] Rubio-Beltran E, Labastida-Ramirez A, Haanes KA, van den Bogaerdt A, Bogers A, Zanelli E (2019). Characterization of binding, functional activity, and contractile responses of the selective 5-HT1F receptor agonist lasmiditan. Br J Pharmacol.

[16] Edvinsson JC, Maddahi A, Christensen IM, Reducha PV, Warfvinge K, Sheykhzade M et al (2022) Lasmiditan and 5-Hydroxytryptamine in the rat trigeminal system; expression, release and interactions with 5-HT1 receptors. J Headache Pain 10.1186/s10194-022-01394-z 10.1186/s10194-022-01394-z PMC8903724 35177004

[17] de Vries T, Villalon CM, MaassenVanDenBrink A (2020) Pharmacological treatment of migraine: CGRP and 5-HT beyond the triptans. Pharmacol Ther 107528 10.1016/j.pharmthera.2020.107528 32173558

[18] De Petrocellis L, Melck D, Bisogno T, Milone A, Di Marzo V (1999). Finding of the endocannabinoid signalling system in Hydra, a very primitive organism: possible role in the feeding response. Neuroscience.

[19] Burggren AC, Shirazi A, Ginder N, London ED (2019). Cannabis effects on brain structure, function, and cognition: considerations for medical uses of cannabis and its derivatives. Am J Drug Alcohol Abuse.

[20] Lowin T, Schneider M, Pongratz G (2019). Joints for joints: cannabinoids in the treatment of rheumatoid arthritis. Curr Opin Rheumatol.

[21] Herkenham M, Lynn AB, Little MD, Johnson MR, Melvin LS, de Costa BR (1990). Cannabinoid receptor localization in brain. Proc Natl Acad Sci U S A.

[22] Castillo PE, Younts TJ, Chavez AE, Hashimotodani Y (2012). Endocannabinoid signaling and synaptic function. Neuron.

[23] Katona I, Sperlagh B, Sik A, Kafalvi A, Vizi ES, Mackie K (1999). Presynaptically located CB1 cannabinoid receptors regulate GABA release from axon terminals of specific hippocampal interneurons. J Neurosci.

[24] Schlicker E, Kathmann M (2001). Modulation of transmitter release via presynaptic cannabinoid receptors. Trends Pharmacol Sci.

[25] Katona I, Sperlagh B, Magloczky Z, Santha E, Kofalvi A, Czirjak S (2000). GABAergic interneurons are the targets of cannabinoid actions in the human hippocampus. Neuroscience.

[26] Caulfield MP, Brown DA (1992). Cannabinoid receptor agonists inhibit ca current in NG108-15 neuroblastoma cells via a pertussis toxin-sensitive mechanism. Br J Pharmacol.

[27] Lauckner JE, Hille B, Mackie K (2005). The cannabinoid agonist WIN55,212-2 increases intracellular calcium via CB1 receptor coupling to Gq/11 G proteins. Proc Natl Acad Sci U S A.

[28] Akerman S, Kaube H, Goadsby PJ (2004). Anandamide is able to inhibit trigeminal neurons using an in vivo model of trigeminovascular-mediated nociception. J Pharmacol Exp Ther.

[29] Akerman S, Holland PR, Goadsby PJ (2007). Cannabinoid (CB1) receptor activation inhibits trigeminovascular neurons. J Pharmacol Exp Ther.

[30] Ruparel NB, Patwardhan AM, Akopian AN, Hargreaves KM (2011). Desensitization of transient receptor potential ankyrin 1 (TRPA1) by the TRP vanilloid 1-selective cannabinoid arachidonoyl-2 chloroethanolamine. Mol Pharmacol.

[31] Engel MA, Izydorczyk I, Mueller-Tribbensee SM, Becker C, Neurath MF, Reeh PW (2011). Inhibitory CB1 and activating/desensitizing TRPV1-mediated cannabinoid actions on CGRP release in rodent skin. Neuropeptides.

[32] Hou M, Uddman R, Tajti J, Kanje M, Edvinsson L (2002). Capsaicin receptor immunoreactivity in the human trigeminal ganglion. Neurosci Lett.

[33] Fischer MJ, Messlinger K (2007). Cannabinoid and vanilloid effects of R(+)-methanandamide in the hemisected meningeal preparation. Cephalalgia.

[34] Hillard CJ, Manna S, Greenberg MJ, DiCamelli R, Ross RA, Stevenson LA (1999). Synthesis and characterization of potent and selective agonists of the neuronal cannabinoid receptor (CB1). J Pharmacol Exp Ther.

[35] Bhatt DK, Gupta S, Jansen-Olesen I, Andrews JS, Olesen J (2013). NXN-188, a selective nNOS inhibitor and a 5-HT1B/1D receptor agonist, inhibits CGRP release in preclinical migraine models. Cephalalgia.

[36] Egea SC, Dickerson IM (2012). Direct interactions between calcitonin-like receptor (CLR) and CGRP-receptor component protein (RCP) regulate CGRP receptor signaling. Endocrinology.

[37] Walker CS, Eftekhari S, Bower RL, Wilderman A, Insel PA, Edvinsson L (2015). A second trigeminal CGRP receptor: function and expression of the AMY1 receptor. Ann Clin Transl Neurol.

[38] Edvinsson L, Edvinsson JCA, Haanes KA (2022). Biological and small molecule strategies in migraine therapy with relation to the calcitonin gene-related peptide family of peptides. Br J Pharmacol.

[39] Walker CS, Raddant AC, Woolley MJ, Russo AF, Hay DL (2018). CGRP receptor antagonist activity of olcegepant depends on the signalling pathway measured. Cephalalgia.

[40] Price TJ, Helesic G, Parghi D, Hargreaves KM, Flores CM (2003). The neuronal distribution of cannabinoid receptor type 1 in the trigeminal ganglion of the rat. Neuroscience.

[41] Hougaard Pedersen S, Maretty L, Ramachandran R, Sibbesen JA, Yakimov V, Elgaard-Christensen R (2016). RNA sequencing of trigeminal ganglia in Rattus Norvegicus after glyceryl Trinitrate infusion with relevance to migraine. PLoS One.

[42] Price TJ, Patwardhan A, Akopian AN, Hargreaves KM, Flores CM (2004). Modulation of trigeminal sensory neuron activity by the dual cannabinoid-vanilloid agonists anandamide, N-arachidonoyl-dopamine and arachidonyl-2-chloroethylamide. Br J Pharmacol.

[43] Richardson JD, Aanonsen L, Hargreaves KM (1998). Antihyperalgesic effects of spinal cannabinoids. Eur J Pharmacol.

[44] Van Der Stelt M, Di Marzo V (2004). Endovanilloids. Putative endogenous ligands of transient receptor potential vanilloid 1 channels. Eur J Biochem.

[45] Akopian AN, Ruparel NB, Jeske NA, Patwardhan A, Hargreaves KM (2009). Role of ionotropic cannabinoid receptors in peripheral antinociception and antihyperalgesia. Trends Pharmacol Sci.

[46] Muller C, Morales P, Reggio PH (2018). Cannabinoid Ligands Targeting TRP Channels. Front Mol Neurosci.

[47] Raboune S, Stuart JM, Leishman E, Takacs SM, Rhodes B, Basnet A (2014). Novel endogenous N-acyl amides activate TRPV1–4 receptors, BV-2 microglia, and are regulated in brain in an acute model of inflammation. Front Cell Neurosci.

[48] Casarotto PC, Terzian AL, Aguiar DC, Zangrossi H, Guimaraes FS, Wotjak CT (2012). Opposing roles for cannabinoid receptor type-1 (CB (1)) and transient receptor potential vanilloid type-1 channel (TRPV1) on the modulation of panic-like responses in rats. Neuropsychopharmacol.

[49] Tognetto M, Amadesi S, Harrison S, Creminon C, Trevisani M, Carreras M (2001). Anandamide excites central terminals of dorsal root ganglion neurons via vanilloid receptor-1 activation. J Neurosci.

[50] Zygmunt PM, Petersson J, Andersson DA, Chuang H, Sorgard M, Di Marzo V (1999). Vanilloid receptors on sensory nerves mediate the vasodilator action of anandamide. Nature.

[51] Smart D, Gunthorpe MJ, Jerman JC, Nasir S, Gray J, Muir AI (2000). The endogenous lipid anandamide is a full agonist at the human vanilloid receptor (hVR1). Br J Pharmacol.

[52] Starkus J, Jansen C, Shimoda LMN, Stokes AJ, Small-Howard AL, Turner H (2019). Diverse TRPV1 responses to cannabinoids. Channels (Austin).

[53] Doherty EM, Fotsch C, Bo Y, Chakrabarti PP, Chen N, Gavva N (2005). Discovery of potent, orally available vanilloid receptor-1 antagonists. Structure-activity relationship of N-aryl cinnamides. J Med Chem.

[54] Summ O, Holland PR, Akerman S, Goadsby PJ (2011). TRPV1 receptor blockade is ineffective in different in vivo models of migraine. Cephalalgia.

[55] Edvinsson L, Haanes KA (2021). Identifying New Antimigraine Targets: Lessons from Molecular Biology. Trends Pharmacol Sci.

