# Supplementary figures and images for: Dual action of the cannabinoid receptor 1 ligand arachidonyl-2′-chloroethylamide on calcitonin gene-related peptide release
Source: J Headache Pain. 2022 Feb 21;23(1):30. doi: 10.1186/s10194-022-01399-8 (PMC8903492; doi:10.1186/s10194-022-01399-8)

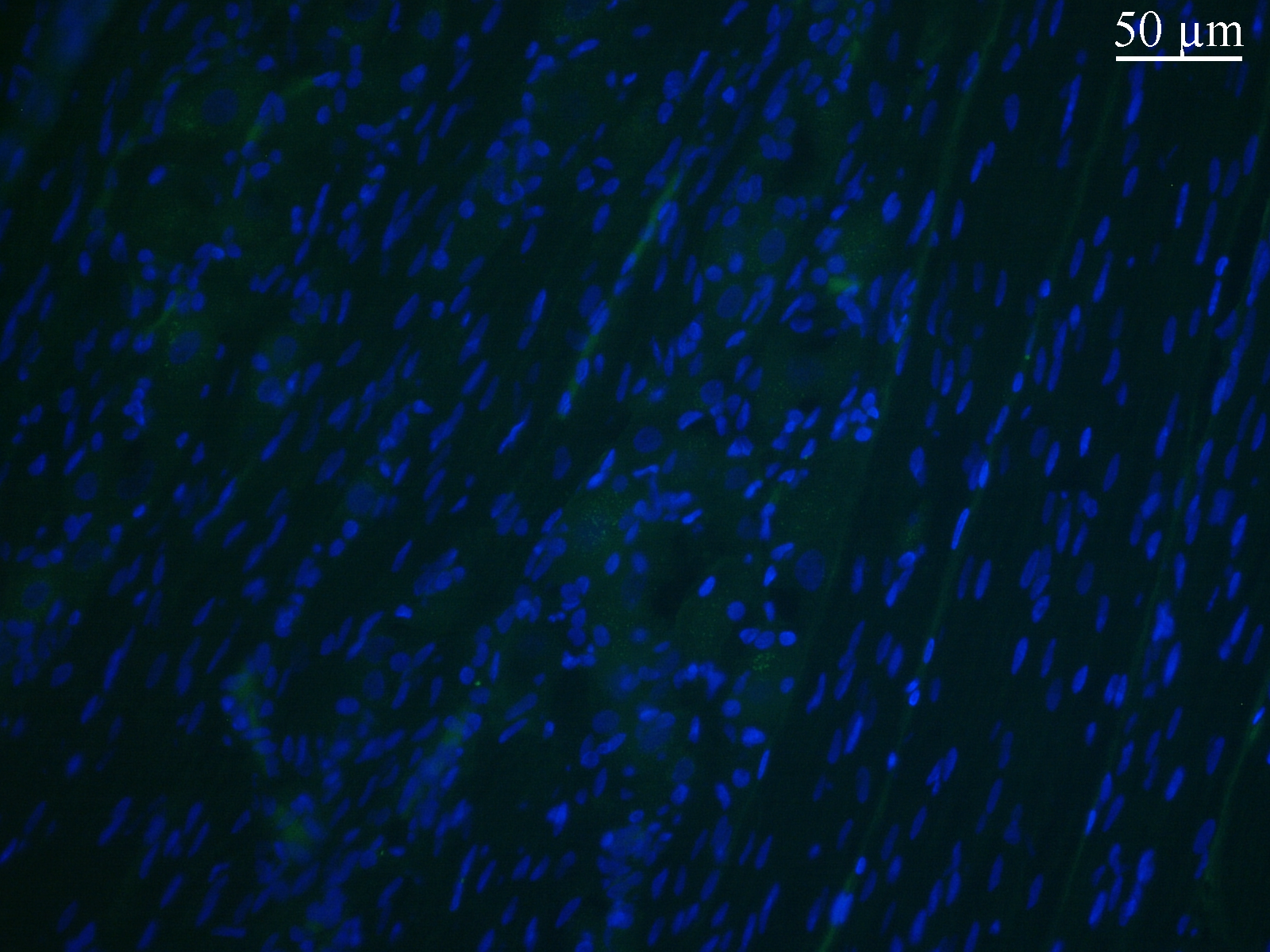

Supplement: Supplementary file 1 — Additional file 1: Supplementary Fig. 1. Negative control for CB1 and CB2. The negative control for the FITC antibody used for CB1 and CB2 IHC. [file 10194_2022_1399_MOESM1_ESM.tif]

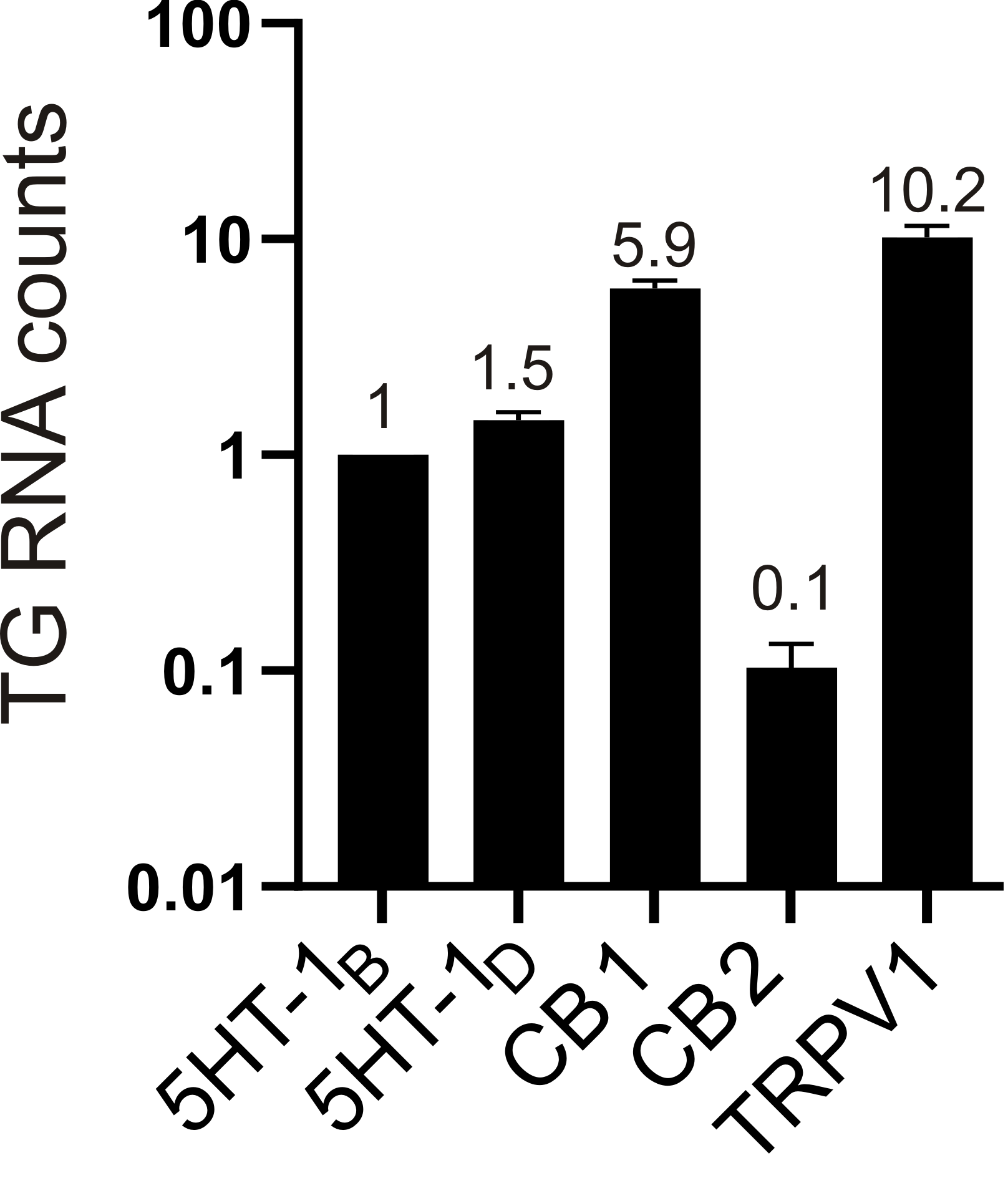

Supplement: Supplementary file 2 — Additional file 2: Supplementary Fig. 2. mRNA expression in the trigeminal ganglion. The average RNA counts of 10 rats in the TG. RNA counts are depicted on a logarithmic scale relative to 5-HT1B and are shown for 5-HT1B; 5-HT1D; CB1; CB2; TRPV1; RAMP1 and CGRP. [file 10194_2022_1399_MOESM2_ESM.tif]
